# Longitudinal Changes in CT-Derived Vertebral Trabecular Attenuation and Psoas Muscle Density After Liver Transplantation for Hepatocellular Carcinoma

**DOI:** 10.3390/medicina62102044

**Published:** 2026-10-21

**Authors:** Mahmut Sahin, Kemal Baris Sarici, Fatih Ahmet Ates, Nurullah Dag, Sami Akbulut

**Affiliations:** 1 Department of Radiology, Inonu University Faculty of Medicine, Malatya 44280, Türkiye; mahmutsahindr@gmail.com (M.S.); fthahmetates@gmail.com (F.A.A.); drndag@icloud.com (N.D.); 2 Department of Surgery and Liver Transplant Institute, Inonu University Faculty of Medicine, Malatya 44280, Türkiye; kemal.sarici@inonu.edu.tr; 3 Department of Biostatistics and Medical Informatics, Inonu University Faculty of Medicine, Malatya 44280, Türkiye

**Keywords:** hepatocellular carcinoma, liver transplantation, computed tomography, vertebral trabecular attenuation, psoas muscle density, psoas muscle index, sarcopenia, bone health

## Abstract

*Background and Objectives*: Hepatocellular carcinoma (HCC) is a major indication for liver transplantation (LT); however, longitudinal musculoskeletal changes detectable by computed tomography (CT) during the first postoperative year remain insufficiently characterized. The aim of this study is to compare baseline CT-derived bone and muscle parameters between patients undergoing LT for HCC and controls and to evaluate longitudinal changes during the first postoperative year. *Materials and Methods:* This retrospective study included 64 patients who underwent LT for HCC and had technically adequate non-contrast abdominal CT examinations at baseline, postoperative day 180 (POD180), and postoperative day 365 (POD365). The control group comprised 64 patients undergoing non-contrast abdominal CT for urinary stone evaluation. CT-derived measures included L1–L4 vertebral trabecular attenuation, psoas muscle area (PMA), the psoas muscle index (PMI), and psoas muscle density (PMD). Repeated-measures analyses and adjusted linear mixed-effects models evaluated longitudinal changes within the HCC cohort. *Results*: At baseline, vertebral trabecular attenuation was higher in the HCC group than in controls (151 ± 36 vs. 137 ± 39, *p* = 0.037), whereas crude PMI, PMA, and PMD did not differ significantly. In hierarchical multivariable analysis, the between-group difference in vertebral attenuation was attenuated after adjustment, while age and baseline PMI remained independently associated with L1–L4 attenuation. In adjusted multivariate analysis, baseline PMD was higher in the HCC group than in controls (51.5 vs. 44.4, *p* < 0.001). Longitudinally, L1–L4 attenuation declined from baseline to POD180 and remained low at POD365 (overall *p* < 0.001), with no difference between POD180 and POD365. PMD also decreased over time (overall *p* < 0.001), whereas PMI and PMA showed no significant change. Adjusted mixed-effects analyses showed that these postoperative reductions persisted after adjustment for age, BMI, albumin, calcium, and ALP. The adjusted PMD model showed an additional decrease between POD180 and POD365 (mean difference, 1.411 HU; 95% CI, 0.065–2.757; Bonferroni-adjusted *p* = 0.037). Older age remained independently associated with lower vertebral attenuation and PMD. *Conclusions*: During the first year after LT for HCC, CT-derived vertebral trabecular attenuation and PMD showed significant longitudinal decreases, while psoas muscle quantity remained relatively stable. Muscle quality and vertebral attenuation may therefore provide earlier imaging signals of post-transplant musculoskeletal deterioration than area-based muscle-quantity measures.

## 1. Introduction

Liver transplantation (LT) remains a cornerstone treatment for carefully selected patients with end-stage liver disease and hepatocellular carcinoma (HCC). As post-transplant survival has improved, clinical attention has increasingly extended beyond short-term graft outcomes to factors influencing postoperative recovery, functional reserve, and long-term prognosis [1,2,3,4,5]. Within this broader perspective, sarcopenia and frailty are increasingly recognized as major clinical manifestations of advanced liver disease rather than merely ancillary nutritional abnormalities [3,6]. Current reviews and meta-analyses indicate that sarcopenia is common among liver transplant candidates and is associated with increased waitlist mortality, poorer post-transplant survival, and a higher risk of postoperative complications and prolonged hospitalization [1,3,7,8]. These considerations are particularly relevant in HCC, where post-transplant outcomes are associated not only with tumor burden but also with host-related body composition characteristics, including skeletal muscle quantity and radiodensity [9,10].

Computed tomography (CT) plays a central role in this field because abdominal imaging is already routinely performed in most LT candidates, enabling opportunistic body composition assessment without additional dedicated examinations [1,11]. CT-based skeletal muscle evaluation, most commonly at the L3 level, is the most widely used imaging approach for sarcopenia assessment in transplant populations [1,7]. Pretransplant CT-derived muscle depletion has been linked to mortality, infection, and prolonged hospitalization after LT [7,11,12,13]. In HCC-specific LT cohorts, this prognostic signal extends beyond muscle quantity alone. Li et al. [9] showed that skeletal muscle density was associated with post-transplant survival, Lu et al. [14] reported adverse effects of myosteatosis and muscle loss, and Lin et al. [10] demonstrated that low skeletal muscle index and low skeletal muscle radiodensity were associated with worse recurrence-free survival in a multicenter HCC cohort. Together, these findings support the clinical relevance of muscle quality as well as muscle quantity in HCC recipients.

Accordingly, exclusive reliance on muscle quantity may be insufficient for defining the musculoskeletal phenotype of LT recipients. Recent literature increasingly emphasizes that muscle quality—typically reflected by CT attenuation or density—captures a distinct biological dimension related to fat infiltration, myosteatosis, and impaired muscle integrity [1,9,12,14]. This distinction matters because patients with similar muscle area may differ substantially in muscle composition and functional reserve. Kaul et al. [15] further showed that malnutrition criteria were more closely related to psoas muscle area than to muscle density, whereas adding CT-derived muscle density improved prediction of length of stay. Collectively, these data support an integrated CT framework in which psoas muscle area, psoas muscle index, and psoas muscle density are interpreted as complementary rather than interchangeable markers.

Bone health has also emerged as another important but underrecognized determinant of post-transplant morbidity. In LT recipients, bone disease reflects the combined effects of chronic liver disease, impaired vitamin metabolism, endocrine dysregulation, reduced physical activity, and post-transplant exposures such as immunosuppressive therapy [16]. Dag et al. [16] showed in a dual-energy X-ray absorptiometry (DXA)-referenced LT cohort that normal bone mineral density was present in only a minority of recipients, whereas osteopenia and osteoporosis accounted for most cases. Opportunistic CT can also provide clinically meaningful information on bone health through vertebral trabecular attenuation measured in Hounsfield units, making it a practical complement to dedicated densitometric techniques [16]. In HCC-specific LT cohorts, Sharma et al. [17] and Toshima et al. [18] both showed that low bone mineral density or osteopenia was independently associated with poorer post-transplant survival or mortality, underscoring the prognostic relevance of bone loss in this setting.

Despite these advances, important gaps remain. Much of the CT-based transplantation literature has focused on single pretransplant measurements, short-term complications, or survival associations based on individual muscle metrics [7,9,11,12,13]. Longitudinal studies are fewer, and when available they usually involve selected populations or limited imaging markers. Recent studies have documented early post-transplant musculoskeletal deterioration in chronic hepatitis B and metabolic dysfunction-associated steatotic liver disease cohorts, but the combined trajectory of bone attenuation and muscle parameters after LT for HCC remains insufficiently defined [19,20]. This gap is clinically important because HCC recipients undergo repeated abdominal imaging during routine follow-up, creating a practical opportunity for integrated monitoring of both bone and muscle phenotypes during the first postoperative year.

Moreover, the literature remains inconsistent not only regarding which musculoskeletal components carry the greatest prognostic relevance in HCC-focused LT cohorts, but also regarding whether HCC itself is associated with a distinctly adverse muscle and bone phenotype. Some studies have linked HCC-related LT cohorts to low muscle mass, myosteatosis, broader body composition abnormalities, or osteopenia, whereas others have not demonstrated uniformly consistent patterns, and sarcopenia alone has not been predictive across all HCC series [9,10,14,17,18,19]. Accordingly, determining whether patients undergoing LT for HCC differ from an appropriate reference population is essential for interpreting the baseline musculoskeletal phenotype, particularly when major demographic determinants such as age and sex are taken into account. In this context, the present study was designed to compare baseline CT-derived bone and muscle parameters between patients undergoing LT for HCC and age- and sex-matched controls, and to characterize longitudinal changes in vertebral trabecular attenuation, psoas muscle area, psoas muscle index, and psoas muscle density during the first year after LT.

## 2. Material and Methods

### 2.1. Study Design and Setting

This study was designed as a single-center, retrospective, observational study with both matched case–control and longitudinal components. The primary objective was to compare baseline CT-derived muscle and bone parameters between patients who underwent LT for HCC and an age- and sex-matched control group. The secondary objective was to evaluate temporal changes in these CT-derived parameters within the HCC cohort across the baseline time point and the first postoperative year.

### 2.2. Study Population

The study population consisted of patients who underwent LT for HCC between 2017 and 2024 at the Liver Transplant Institute, Inonu University, Malatya, Türkiye. Patients were eligible for inclusion if non-contrast abdominal CT examinations suitable for musculoskeletal assessment were available at all three predefined time points: the baseline examination, defined as the last available CT obtained within the month before transplantation, approximately postoperative day 180 (POD180), and approximately postoperative day 365 (POD365). A total of 64 patients met these imaging eligibility criteria and were included in the HCC cohort. The control group consisted of 64 individuals matched to the HCC cohort according to age and sex. Controls were selected from subjects who underwent non-contrast abdominal CT using a urinary stone protocol because of urinary system complaints. Individuals with known malignancy or systemic disease were not included in the control group. In the control group, CT-based muscle and bone measurements were performed at a single time point using the available examination, which was considered the baseline CT for cross-sectional comparisons.

### 2.3. Study Variables and Assessed Parameters

The evaluated variables included demographic, anthropometric, laboratory, and CT-derived musculoskeletal parameters. Demographic variables comprised age and sex, while anthropometric variables included height, weight, and body mass index (BMI). CT-derived parameters included mean vertebral trabecular attenuation across the measurable lumbar vertebral levels (L1–L4), total psoas muscle area (PMA), psoas muscle index (PMI), and psoas muscle density (PMD). In the HCC cohort, baseline laboratory evaluation was based on the last available measurements obtained before liver transplantation. In the control group, baseline laboratory evaluation was based on measurements obtained on the day of CT examination or, when unavailable, the closest available values immediately preceding CT. In the control group, laboratory measurements and CT examinations were performed on the same day for all included subjects. Laboratory variables included albumin, total protein, calcium, phosphorus, hemoglobin, white blood cell (WBC) count, neutrophil count, lymphocyte count, platelet count, red cell distribution width (RDW), mean platelet volume (MPV), and alkaline phosphatase (ALP).

### 2.4. Inclusion and Exclusion Criteria

For the HCC cohort, the inclusion criteria were as follows: LT performed for HCC, availability of technically adequate non-contrast abdominal CT examinations at the predefined study time points, and image quality sufficient for reliable bone and muscle measurements. Patients were excluded if one or more of the required CT examinations were unavailable, if longitudinal imaging assessment could not be completed, or if image quality was inadequate for quantitative analysis. Additional exclusion criteria were applied to ensure measurement reliability. Examinations were excluded in the presence of marked focal bone lesions at the lumbar vertebral levels, severe degenerative change, vertebral compression fracture, diffuse sclerotic change, or imaging artifacts that could compromise attenuation measurements. For control subjects, eligibility required age and sex compatibility with the HCC group, absence of known malignancy or systemic disease, and availability of a technically adequate non-contrast abdominal CT examination.

### 2.5. CT Acquisition Protocol

All CT examinations had been acquired previously for clinical indications, and no additional imaging was performed specifically for this study. Both HCC patients and controls were evaluated using non-contrast abdominal CT images. Examinations were performed on multidetector CT systems, including a 256-slice Somatom Definition scanner (Siemens Healthineers, Erlangen, Germany), and a 64-slice Aquilion CT scanner (Canon Medical Systems, Otawara, Japan). CT acquisition was performed according to routine abdominal imaging principles. The general protocol included a tube voltage of 120 kVp, automatic tube current modulation, a 512 × 512 reconstruction matrix, and a standard soft-tissue reconstruction algorithm. Axial images and sagittal multiplanar reformations were used for quantitative analysis. Particular attention was paid to the use of standardized anatomical landmarks and to maintaining comparable technical conditions across serial examinations in the HCC cohort whenever possible. All measurements were obtained from non-contrast CT examinations using standardized anatomical landmarks and ROI placement criteria.

### 2.6. Vertebral Trabecular Attenuation Measurements

Vertebral trabecular bone attenuation was used as the CT-based bone parameter. Measurements were performed on sagittal reformatted images by placing a circular or ovoid region of interest (ROI) within the central trabecular compartment of the lumbar vertebral body. During ROI placement, cortical bone, vertebral endplates, basivertebral venous structures, focal lytic or sclerotic lesions, marked degenerative changes, and visible artifacts were carefully excluded. Whenever technically feasible, attenuation measurements were obtained separately at the L1, L2, L3, and L4 vertebral levels. For each level, the mean attenuation value was recorded in Hounsfield units (HU). To reduce potential biological and technical variability across vertebral levels, the mean value of all measurable lumbar levels was used as the representative vertebral attenuation parameter for statistical analysis. Vertebral levels affected by focal lesions, marked degenerative changes, compression fractures, or relevant artifacts were excluded from the mean attenuation measurement at the corresponding examination.

### 2.7. PMA and PMI Measurements

Psoas muscle quantity was assessed at the level of the third lumbar vertebra (L3), which served as the anatomical reference level for CT-based psoas muscle analysis. On axial CT images, the section in which both transverse processes were clearly visualized was selected. The right and left psoas muscles were manually contoured separately using freehand ROI tracing, and the cross-sectional area of each muscle was measured in square centimeters (cm^2^). The sum of the right and left psoas areas was defined as the total psoas muscle area. To account for differences in body size, the PMI was calculated by dividing the total psoas muscle area at the L3 level by the square of patient height in meters. PMI values were recorded in cm^2^/m^2^.

### 2.8. PMD Measurements

Muscle quality was assessed using psoas muscle density (PMD) at the L3 level. Regions of interest were placed within the psoas muscle boundaries while carefully avoiding adjacent fat planes and non-muscular structures. The right and left psoas muscles were measured separately, and their mean attenuation value, expressed in Hounsfield units, was recorded as PMD.

### 2.9. Image Analysis and Measurement Standardization

All image analyses were performed using the institutional IDS7 PACS (Sectra Workstation IDS7, version 28.1.8.10614; Sectra AB, Linköping, Sweden) on standard diagnostic radiology workstations. Measurements were carried out by radiologists experienced in abdominal and musculoskeletal imaging. Before the formal analysis, a standardized approach was defined for anatomical level selection, ROI placement, and exclusion of potentially confounding image findings. In cases with focal vertebral abnormality, marked degenerative change, or technical uncertainty affecting measurement reliability, the relevant vertebral level was excluded from analysis. When necessary, consensus review was used to ensure consistent application of measurement criteria across examinations. Interobserver reproducibility was assessed in a randomly selected subset of 13 patients by two radiologists with 5 and 15 years of radiology experience, respectively. Both readers performed the measurements independently using the same predefined anatomical landmarks and ROI placement criteria. Agreement was evaluated using intraclass correlation coefficients (ICCs).

### 2.10. Study Outcomes

The primary study outcome was the difference in CT-derived bone and muscle parameters between the HCC group at the baseline time point and the age- and sex-matched control group. The secondary outcome was the longitudinal change in these CT-derived parameters within the HCC cohort across the three predefined time points: baseline, POD180, and POD365.

### 2.11. Study Protocol, Ethical Considerations, and Funding

The study was conducted in accordance with the ethical principles of the Declaration of Helsinki, including its 2024 revision, and with all applicable institutional and national regulations governing research involving human participants [21]. Ethical approval was obtained from the Inonu University Health Sciences Scientific Research Ethics Committee (session date: 21 April 2026; session number: 8; decision number: 2026/10040). Given the retrospective design of the study and the exclusive use of archived radiological images obtained during routine clinical care, no additional imaging or intervention was performed, and informed consent was not required. The study was financially supported by the Inonu University Scientific Research Projects Coordination Unit (Grant ID: TSA-2026-5043). The study was designed, conducted, analyzed, and reported in accordance with the STROBE (Strengthening the Reporting of Observational Studies in Epidemiology) guidelines to promote transparency, reproducibility, and methodological rigor.

### 2.12. Statistical Analysis

Statistical analyses were performed using IBM SPSS Statistics for Windows, version 25.0 (IBM Corp., Armonk, NY, USA). Graphs were generated using GraphPad Prism version 10.6.1 for Windows (GraphPad Software, Boston, MA, USA). Statistical analyses were structured according to the two main components of the study: (1) cross-sectional analyses comparing the HCC and control groups at the baseline time point and (2) longitudinal analyses within the HCC cohort across baseline, POD180, and POD365. The distribution of continuous variables was assessed using the Shapiro–Wilk test. Continuous variables were summarized as means ± standard deviations or medians (interquartile range), as appropriate, and categorical variables were presented as numbers and percentages.

For the cross-sectional analyses, continuous variables were compared using Student’s *t*-test for normally distributed data or the Mann–Whitney U test for non-normally distributed data. Categorical variables were compared using the chi-square test or Fisher’s exact test, as appropriate. Univariable linear regression analyses were performed to assess crude associations between selected demographic, clinical, biochemical, and CT-derived parameters and baseline L1–L4 attenuation. Variables considered clinically relevant or associated with baseline L1–L4 attenuation in univariable analyses were then entered into a hierarchical multivariable linear regression model with baseline L1–L4 attenuation as the dependent variable. Model performance was summarized using R^2^, adjusted R^2^, ΔR^2^, and the *p* value for ΔR^2^. As an additional multivariate analysis of the cross-sectional component, a multivariate analysis of covariance (MANCOVA) was performed to assess whether the overall baseline CT-derived musculoskeletal profile differed between the HCC and control groups after adjustment for potential confounders. Baseline L1–L4 vertebral trabecular attenuation, baseline PMI, and baseline PMD were entered jointly as dependent variables. Study group and sex were entered as fixed factors, while age, BMI, albumin, calcium, and ALP were entered as covariates, and the group-by-sex interaction was also tested. Multivariate significance was assessed using Pillai’s trace. When the overall multivariate group effect was significant, follow-up univariate analyses and Bonferroni-adjusted pairwise comparisons of estimated marginal means were examined for each dependent variable. Effect sizes were reported as partial eta-squared (partial η^2^).

For the longitudinal analyses within the HCC cohort, repeated measurements obtained at baseline, POD180, and POD365 were analyzed using one-way repeated-measures analysis of variance (ANOVA) for normally distributed variables. Adjusted linear mixed-effects models included time as a fixed effect and patient as a random effect. Age and baseline BMI, albumin, calcium, and ALP were included as covariates. The assumption of sphericity was evaluated using Mauchly’s test, and the Greenhouse–Geisser correction was applied when necessary. For significant overall time effects, Bonferroni-adjusted pairwise comparisons were performed to determine differences between individual time points. For variables not meeting parametric assumptions, the Friedman test was used, followed by Bonferroni-adjusted post hoc pairwise comparisons. No between-group repeated-measures model was constructed because the longitudinal component of the study included only the HCC cohort. Interobserver agreement was evaluated using a two-way random-effects, absolute-agreement, single-measure ICC. A two-sided *p* value < 0.05 was considered statistically significant.

## 3. Results

### 3.1. Cross-Sectional Analyses Comparing the HCC and Control Groups

#### 3.1.1. Baseline Demographic, Laboratory, and Preoperative CT-Derived Characteristics

Table 1 shows the baseline demographic, laboratory, and preoperative CT-derived characteristics of the HCC and control groups. A total of 128 participants were included in the cross-sectional analysis, comprising 64 patients in the HCC group and 64 age- and sex-matched controls. Sex distribution was comparable between the groups (male: 92.2% vs. 90.6%, *p* = 0.752). Age and height were also similar (both *p* > 0.05). In contrast, the HCC group had significantly lower body weight and BMI than the control group (weight: *p* = 0.012; BMI: *p* = 0.030). Regarding laboratory parameters, the HCC group showed significantly lower albumin (*p* < 0.001), total protein (*p* = 0.014), calcium (*p* = 0.001), hemoglobin (*p* = 0.011), WBC (*p* < 0.001), neutrophil count (*p* < 0.001), lymphocyte count (*p* < 0.001), and platelet count (*p* < 0.001). By contrast, RDW (*p* < 0.001), MPV (*p* < 0.001), and ALP (*p* < 0.001) were significantly higher in the HCC group. No significant between-group differences were observed for phosphorus (*p* = 0.149).

#### 3.1.2. Between-Group Differences in Baseline CT-Derived Bone and Muscle Parameters

**Figure 1:**
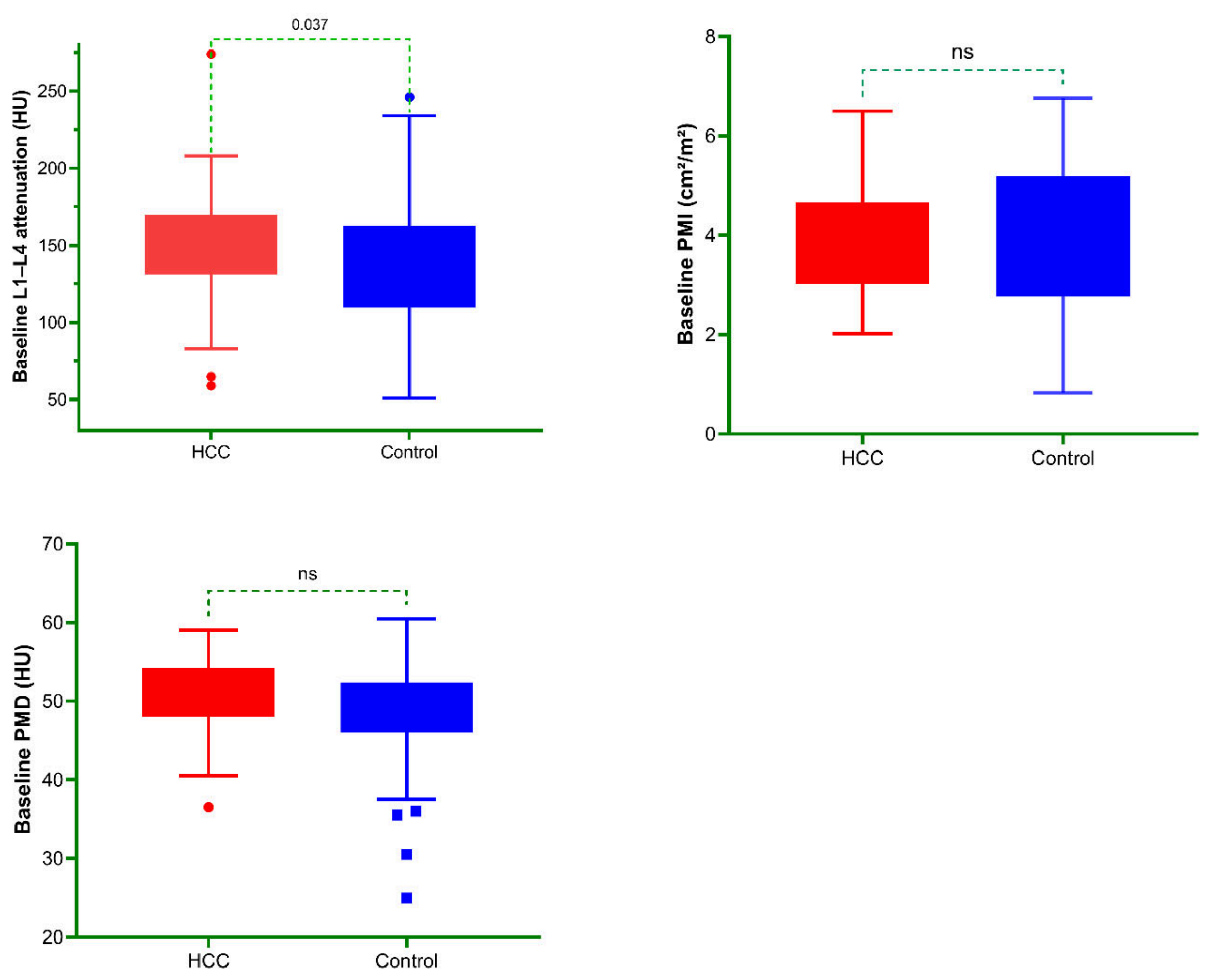
Between-group comparisons of selected baseline CT-derived bone and muscle parameters in the HCC and control groups. Box-and-whisker plots showing baseline L1–4 vertebral trabecular attenuation, baseline psoas muscle index, and baseline psoas muscle density in the HCC and control groups. *p* values represent between-group comparisons for each parameter. ns: not statistically significant.

Table 1 shows that baseline L1–L4 vertebral trabecular attenuation was significantly higher in the HCC group than in the control group (151 ± 36 vs. 137 ± 39, *p* = 0.037). In contrast, no significant between-group differences were found for baseline PMI (*p* = 0.634), PMA (*p* = 0.442), or PMD (*p* = 0.108). These between-group differences in baseline CT-derived bone and muscle parameters are illustrated in Figure 1. These findings indicate that the crude between-group difference was primarily observed in vertebral trabecular attenuation rather than in CT-derived psoas muscle parameters. Interobserver reproducibility was excellent in the 13-patient subset. ICCs for vertebral attenuation were 0.962, 0.955, and 0.948 at baseline, POD180, and the one-year follow-up, respectively. Corresponding ICCs were 0.959, 0.948, and 0.970 for PMA and 0.971, 0.984, and 0.982 for PMD.

#### 3.1.3. Univariable Linear Associations of Clinical and CT-Derived Parameters with Baseline L1–L4 Attenuation

**Figure 2:**
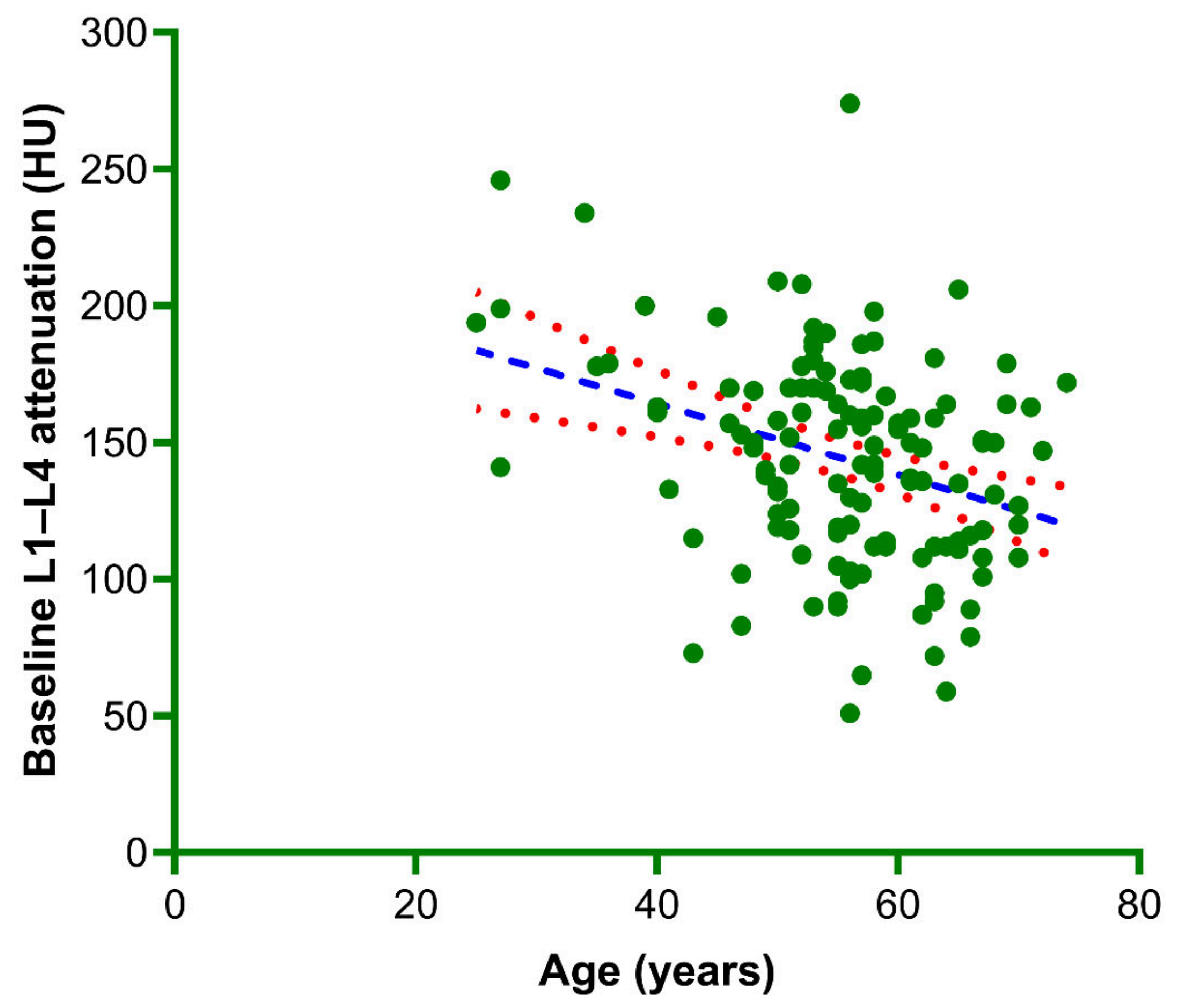
Association between age and baseline L1–L4 vertebral trabecular attenuation. Scatter plot with fitted linear regression line and 95% confidence bands showing a significant inverse association between age and baseline L1–L4 attenuation. The blue dashed line represents the fitted linear regression line, and the red dotted lines indicate the 95% confidence limits.

**Figure 3:**
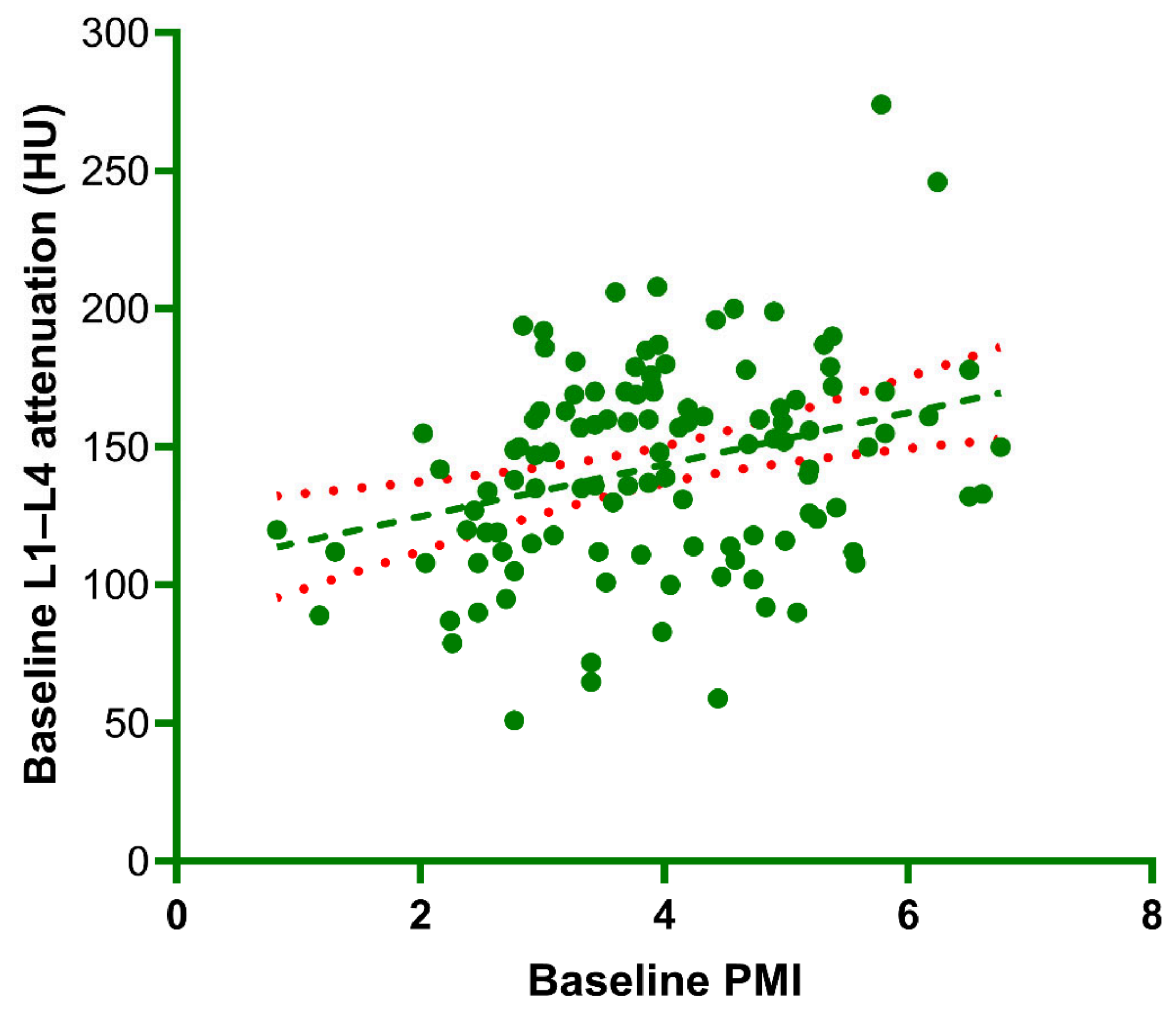
Association between baseline psoas muscle index and baseline L1–L4 vertebral trabecular attenuation. Scatter plot with fitted linear regression line and 95% confidence bands showing a significant positive association between baseline PMI and baseline L1–L4 attenuation. The green dashed line represents the fitted linear regression line, and the red dotted lines indicate the 95% confidence limits.

Table 2 shows the crude linear associations of selected demographic, clinical, biochemical, and CT-derived parameters with baseline L1–L4 attenuation in the univariable linear regression analyses. Study group (control vs. HCC) was significantly associated with baseline L1–L4 attenuation (B = −14.094, 95% CI −27.326 to −0.862, *p* = 0.037; R^2^ = 0.034). Age showed a significant inverse association with baseline L1–L4 attenuation (B = −1.301, 95% CI −1.968 to −0.634, *p* < 0.001; R^2^ = 0.106), indicating that vertebral attenuation decreased as age increased (Figure 2). Baseline L1–L4 attenuation was also positively associated with baseline PMI (B = 9.390, 95% CI 3.933 to 14.847, *p* = 0.001; R^2^ = 0.094) (Figure 3) and baseline PMD (B = 1.423, 95% CI 0.244 to 2.602, *p* = 0.018; R^2^ = 0.043). Sex, BMI, albumin, calcium, and ALP were not significantly associated with baseline L1–L4 attenuation in the univariable analyses (all *p* > 0.05). These findings represent crude associations and should not be interpreted as equivalent to the adjusted estimates obtained in the multivariable model.

#### 3.1.4. Hierarchical Multivariable Linear Regression Analysis of Baseline L1–L4 Attenuation

**Figure 4:**
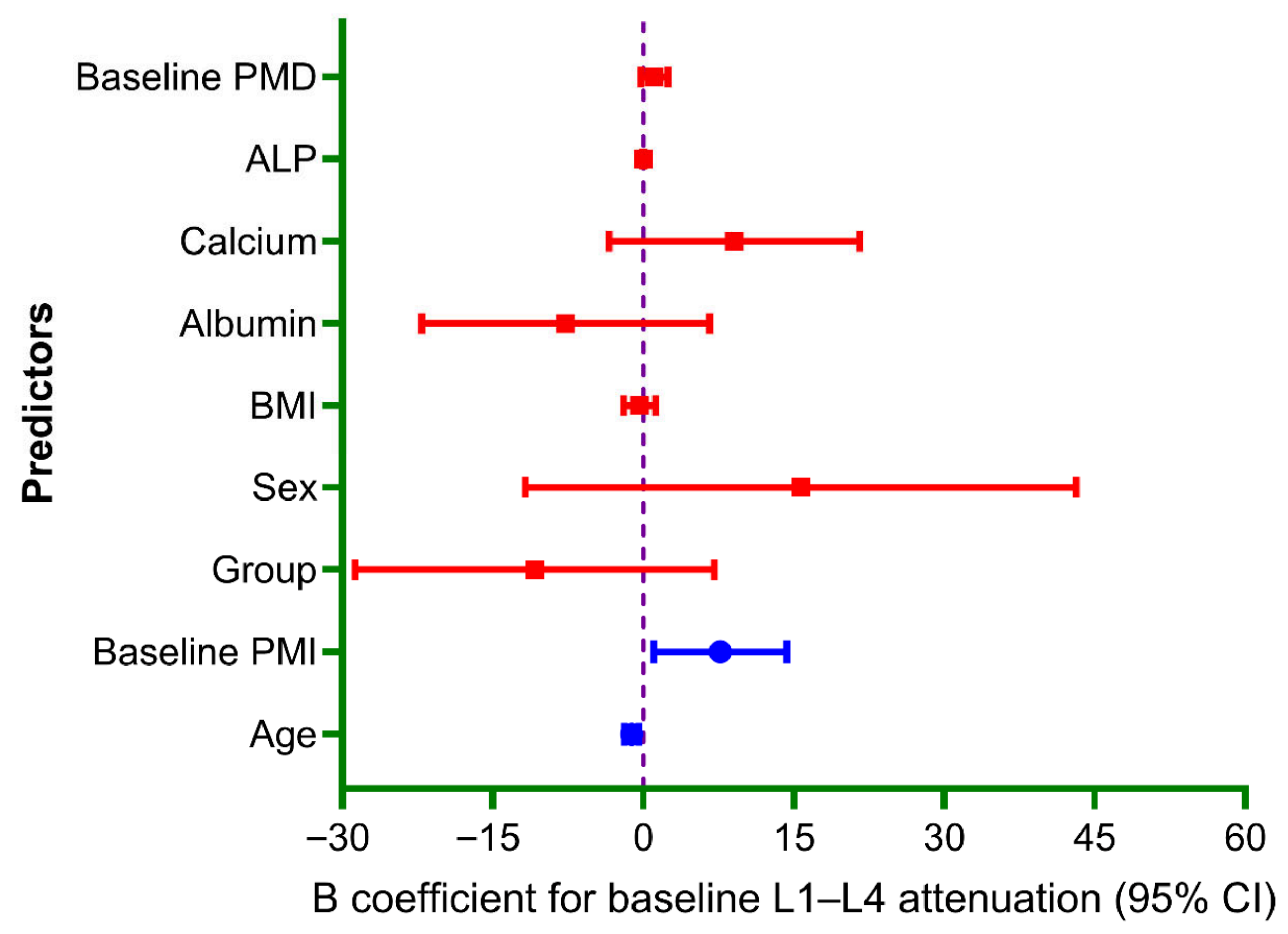
Forest plot of the final multivariable linear regression model for baseline L1–L4 vertebral trabecular attenuation. Forest plot showing unstandardized regression coefficients (B) and 95% confidence intervals for factors included in the final multivariable model of baseline L1–L4 attenuation. Blue indicates statistically significant associations (*p* < 0.05), whereas red indicates nonsignificant associations. The purple dashed vertical line marks B = 0.

Table 3 shows the results of the hierarchical multivariable linear regression analysis conducted in the complete-case sample of 109 participants to evaluate factors associated with the crude between-group difference in vertebral trabecular attenuation, with baseline L1–L4 attenuation entered as the dependent variable. The final multivariable model is summarized in Figure 4. In Model 1, which was estimated in the same complete-case sample used for all hierarchical models, study group was significantly associated with baseline L1–L4 attenuation (B = −16.723, *p* = 0.021; R^2^ = 0.049). After adjustment for age, sex, and BMI, the model improved significantly (Model 2: ΔR^2^ = 0.126, *p* = 0.002), and age was independently and inversely associated with baseline L1–L4 attenuation (B = −1.375, *p* < 0.001), whereas the group effect remained significant (B = −16.107, *p* = 0.022).

The subsequent addition of albumin, calcium, and ALP did not significantly improve model performance (Model 3: ΔR^2^ = 0.019, *p* = 0.492), although the group effect remained statistically significant (B = −19.098, *p* = 0.031). In the final model, inclusion of CT-derived muscle parameters significantly improved the explained variance (Model 4: ΔR^2^ = 0.069, *p* = 0.012; final model R^2^ = 0.263). In this fully adjusted model, the association between study group and baseline L1–L4 attenuation was no longer significant (B = −10.842, *p* = 0.232), whereas age (B = −1.187, *p* = 0.002) and baseline PMI (B = 7.654, *p* = 0.024) remained independently associated with vertebral attenuation. In practical terms, each 1-year increase in age was associated with an approximately 1.2-HU decrease in baseline L1–L4 attenuation, and each 1 cm^2^/m^2^ increase in baseline PMI was associated with an approximately 7.7-HU increase in vertebral attenuation. Baseline PMD was not independently associated with L1–L4 attenuation (B = 1.090, *p* = 0.113). Taken together, the crude between-group difference in baseline vertebral trabecular attenuation was attenuated after adjustment, while age and baseline PMI remained independently associated with vertebral trabecular attenuation.

#### 3.1.5. Multivariate Analysis of Baseline CT-Derived Musculoskeletal Profile

Table 4 shows the results of the multivariate analysis evaluating whether the baseline CT-derived musculoskeletal profile differed between the HCC and control groups after adjustment for demographic and biochemical covariates. A MANCOVA was performed in the complete-case sample of 109 participants, with baseline L1–L4 attenuation, baseline PMI, and baseline PMD entered jointly as dependent variables. The overall multivariate effect of study groups was significant (Pillai’s trace = 0.138, F = 5.238, *p* = 0.002, partial η^2^ = 0.138), indicating that the adjusted baseline musculoskeletal profile differed between the HCC and control groups. No significant group-by-sex interaction was observed (Pillai’s trace = 0.042, F = 1.438, *p* = 0.236, partial η^2^ = 0.042).

In follow-up univariate analyses, the adjusted group difference was not significant for baseline L1–L4 attenuation (F = 1.356, *p* = 0.247, partial η^2^ = 0.013) or baseline PMI (F = 2.542, *p* = 0.114, partial η^2^ = 0.025). In contrast, baseline PMD remained significantly higher in the HCC group than in the control group (F = 14.993, *p* < 0.001, partial η^2^ = 0.130). Estimated marginal means showed adjusted PMD values of 51.5 HU in the HCC group and 44.4 HU in the control group, corresponding to an adjusted mean difference of 7.1 HU (95% CI, 3.5–10.7; Bonferroni-adjusted *p* < 0.001). These findings indicate that, after adjustment for age, sex, BMI, albumin, calcium, and ALP, the between-group difference in the overall CT-derived musculoskeletal profile was driven primarily by psoas muscle density rather than by vertebral trabecular attenuation or PMI.

### 3.2. Longitudinal Analyses Within the HCC Cohort

#### 3.2.1. Primary Repeated-Measures Analysis

Table 5 shows the longitudinal changes in CT-derived bone and muscle parameters within the HCC cohort of 64 patients. L1–L4 vertebral trabecular attenuation changed significantly over time. Because the assumption of sphericity was violated, the Greenhouse–Geisser-corrected result was considered, which showed a significant overall time effect (F = 54.022, *p* < 0.001, partial η^2^ = 0.462). Mean L1–L4 attenuation decreased from 151.1 ± 36.4 HU at baseline to 121.6 ± 35.0 HU at POD180 and remained low at 122.3 ± 37.2 HU at POD365. Bonferroni-adjusted pairwise comparisons demonstrated significant reductions from baseline to POD180 and from baseline to POD365 (both *p* < 0.001), whereas no significant difference was observed between POD180 and POD365 (*p* = 1.000).

In contrast, no significant longitudinal change was observed for PMI or PMA. Mean PMI values were 3.91 ± 1.06 cm^2^/m^2^ at baseline, 3.72 ± 1.08 cm^2^/m^2^ at POD180, and 3.93 ± 1.16 cm^2^/m^2^ at POD365 (F = 2.005, *p* = 0.139, partial η^2^ = 0.031). Mean PMA values were 11.57 ± 3.34 cm^2^ at baseline, 11.07 ± 3.52 cm^2^ at POD180, and 11.67 ± 3.74 cm^2^ at POD365 (F = 1.788, *p* = 0.171, partial η^2^ = 0.028). Bonferroni-adjusted pairwise comparisons showed no significant differences between any time points for either PMI or PMA.

**Figure 5:**
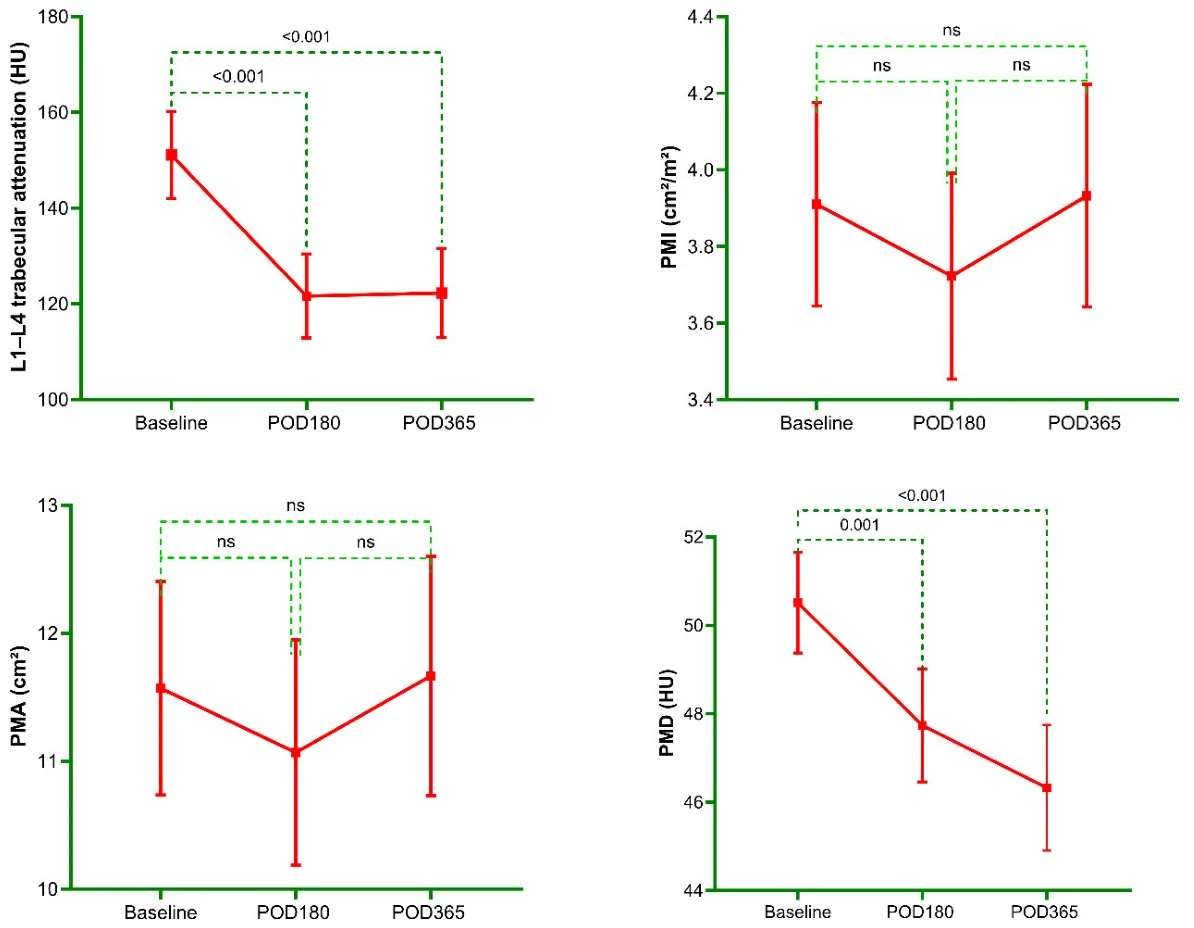
Longitudinal changes in CT-derived vertebral trabecular attenuation and psoas muscle parameters within the HCC cohort. ns, not statistically significant.

PMD also changed significantly over time (Friedman χ^2^ = 22.842, df = 2, *p* < 0.001). Mean PMD decreased from 50.52 ± 4.57 HU at baseline to 47.74 ± 5.12 HU at POD180 and to 46.33 ± 5.70 HU at POD365. Post hoc Wilcoxon signed-rank tests showed significant reductions from baseline to POD180 and from baseline to POD365 (both *p* < 0.001). Although the comparison between POD180 and POD365 yielded a nominal *p* value of 0.020, it did not meet the Bonferroni-adjusted significance threshold of *p* < 0.0167. The longitudinal trajectories of L1–L4 vertebral trabecular attenuation, PMI, PMA, and PMD across baseline, POD180, and POD365 are illustrated in Figure 5.

#### 3.2.2. Adjusted Linear Mixed-Effects Analysis

As an adjusted sensitivity analysis, linear mixed-effects models were performed for L1–L4 vertebral trabecular attenuation and PMD. Time point was entered as a fixed effect, patient identity was used to account for within-patient repeated measurements, and age, BMI, albumin, calcium, and ALP were included as covariates.

Table 6 shows the fixed effects and adjusted pairwise comparisons from the linear mixed-effects models for L1–L4 vertebral trabecular attenuation and PMD. For L1–L4 vertebral trabecular attenuation, the adjusted mixed-effects model confirmed a significant overall time effect (F = 50.761, *p* < 0.001). Adjusted mean L1–L4 attenuation decreased from 151.1 HU at baseline to 121.6 HU at POD180 and remained low at 122.3 HU at POD365. Bonferroni-adjusted pairwise comparisons showed significant reductions from baseline to POD180 (mean difference, 29.437 HU; 95% CI, 22.091–36.784; *p* < 0.001) and from baseline to POD365 (mean difference, 28.812 HU; 95% CI, 19.715–37.910; *p* < 0.001), with no significant difference between POD180 and POD365 (mean difference, −0.625 HU; 95% CI, −7.926 to 6.676; *p* = 1.000). Among the covariates, age was independently associated with lower L1–L4 attenuation; each 1-year increase in age was associated with an approximately 1.12-HU decrease in vertebral attenuation (B = −1.125, 95% CI, −2.024 to −0.226; *p* = 0.015). BMI, albumin, calcium, and ALP were not significantly associated with L1–L4 attenuation.

For PMD, the adjusted mixed-effects model also demonstrated a significant overall time effect (F = 17.838, *p* < 0.001). Adjusted mean PMD decreased from 50.5 HU at baseline to 47.7 HU at POD180 and to 46.3 HU at POD365. Pairwise comparisons showed significant reductions from baseline to POD180 (mean difference, 2.780 HU; 95% CI, 1.211–4.349; Bonferroni-adjusted *p* < 0.001) and from baseline to POD365 (mean difference, 4.191 HU; 95% CI, 2.459–5.922; Bonferroni-adjusted *p* < 0.001). A smaller but statistically significant additional decrease was observed between POD180 and POD365 (mean difference, 1.411 HU; 95% CI, 0.065–2.757; Bonferroni-adjusted *p* = 0.037). Age was independently associated with lower PMD; each 1-year increase in age was associated with an approximately 0.113-HU decrease in PMD (B = −0.113, 95% CI, −0.223 to −0.003; *p* = 0.045). BMI, albumin, calcium, and ALP were not significantly associated with PMD. Taken together, these adjusted longitudinal models showed that the observed postoperative reductions in vertebral trabecular attenuation and PMD persisted after adjustment for the included covariates, while older age remained independently associated with lower vertebral attenuation and lower muscle density.

## 4. Discussion

The present study showed that the first postoperative year after LT for HCC was characterized by a substantial decline in vertebral trabecular attenuation and PMD, whereas no significant longitudinal changes were observed in PMA or PMI. The crude baseline difference in vertebral attenuation between the HCC and control groups was no longer significant after multivariable adjustment, whereas adjusted baseline PMD remained higher in the HCC group. Although the groups were matched for age and sex, differences in BMI and laboratory characteristics indicate residual clinical heterogeneity. The discrepancy between crude and adjusted PMD comparisons likely reflects the influence of these covariates and should therefore be interpreted cautiously. These findings indicate that the dominant postoperative change involved tissue composition and trabecular bone attenuation rather than overt reduction in psoas size. Most previous LT studies have evaluated a single pretransplant muscle measurement and related it to mortality or postoperative complications. Van Vugt et al. [7] demonstrated that low CT-defined skeletal muscle mass was associated with adverse waitlist and post-LT outcomes, while Akabane et al. [8] confirmed an association between pretransplant sarcopenia and post-LT mortality in an updated meta-analysis. In HCC, Beumer et al. [22] showed that low skeletal muscle index was associated with poorer survival after LT beyond the Milan criteria, and Guo et al. [23] linked sarcopenia to mortality and recurrence across different HCC treatments. These studies establish the clinical importance of muscle depletion but do not clarify whether muscle quantity, muscle quality, and bone attenuation follow the same postoperative trajectory. The present study addresses this gap by evaluating all three dimensions serially at predefined time points. Its principal contribution is the observation that PMD and vertebral trabecular attenuation may decline despite the absence of significant changes in psoas dimensions, suggesting that conventional area-based measures alone may underestimate early postoperative musculoskeletal vulnerability.

The present study detected a significant longitudinal change in PMD, whereas no significant changes were observed in PMA or PMI, supporting the concept that muscle quality and muscle quantity are biologically related but not interchangeable. PMA and PMI reflect the amount of muscle tissue, whereas PMD reflects its radiological composition. Reduced CT attenuation is commonly interpreted as greater intramuscular lipid infiltration and myosteatosis, although edema, fibrosis, hydration status, glycogen content, and technical acquisition factors may also affect measured density. Huang et al. [24] defined myosteatosis using reduced skeletal muscle radiodensity and reported that pretransplant myosteatosis was associated with higher post-LT mortality and complication rates. Li et al. [9] specifically evaluated HCC recipients and found that lower skeletal muscle density was independently associated with poorer post-LT survival, whereas the prognostic performance of quantity-based indices was less consistent. Lu et al. [14] showed that myosteatosis further worsened outcomes among sarcopenic male HCC recipients and that postoperative muscle loss was associated with inferior survival in selected subgroups. Lin et al. [10] subsequently demonstrated in a multicenter cohort that both low skeletal muscle index and low radiodensity contributed prognostic information, indicating that the two measurements capture different aspects of host reserve. The present observation of declining PMD without corresponding significant longitudinal changes in PMA or PMI is consistent with this distinction. Clinically, this pattern suggests that deterioration in muscle composition may precede measurable loss of muscle quantity. Kaul et al. [15] found that formal malnutrition criteria correlated more closely with psoas area than with density, but adding psoas density improved prediction of hospital length of stay. Lepour et al. [25] also found low muscle density to be prognostically informative, although functional performance was more strongly associated with outcome after adjustment. Collectively, these findings suggest that PMD may provide a more sensitive imaging signal of compositional change than area-based measures during the first postoperative year, but it should be interpreted together with muscle quantity and objective functional assessment rather than as a stand-alone definition of sarcopenia.

The present study also demonstrated a marked reduction in mean L1–L4 vertebral trabecular attenuation by POD180, followed by persistence of the lower level at POD365. This longitudinal decline should be distinguished from the baseline case–control comparison, because the crude difference in vertebral attenuation was substantially attenuated after adjustment for age and muscle-related variables. The longitudinal postoperative decline in vertebral attenuation was more consistent than the baseline between-group difference, which was no longer significant in the fully adjusted model. Monegal et al. [26] prospectively observed that bone mass decreased during the first six months after LT and subsequently recovered at different rates at the lumbar spine and femoral neck. Ninkovic et al. [27] likewise described early skeletal loss after transplantation. These longitudinal observations are consistent with the timing identified in the present study. Butin et al. [28] reported a marked increase in vertebral fracture prevalence during the first postoperative year, providing clinical context for the early attenuation decline. Almusrea et al. [29] similarly found that vertebral fracture incidence was highest during the first year after LT. Longer-term studies indicate that risk does not resolve uniformly: Abate et al. [30] showed that selected recipients continued to experience progression of bone fragility beyond the first postoperative year, while Zavatta et al. [31] documented a substantial burden of vertebral fractures in a modern LT cohort. In HCC-specific populations, Sharma et al. [17] found lower CT-derived bone density to be independently associated with post-LT mortality, and Toshima et al. [18] reported an association between preoperative osteopenia and poorer survival after living-donor LT. These prognostic studies support the clinical relevance of CT-derived bone measurements but do not directly establish that attenuation decline represents osteoporosis. Accordingly, the present findings should be interpreted as evidence of early deterioration in trabecular attenuation that may identify recipients requiring formal bone-health evaluation.

The present study identified concurrent deterioration in PMD and vertebral attenuation, supporting interpretation of postoperative changes within an integrated muscle–bone unit rather than as two unrelated complications. Dag et al. [19] also reported parallel reductions in psoas measurements and L1–L4 attenuation after LT in patients with chronic hepatitis B, although PMA and PMI decreased in that cohort, whereas muscle quantity remained stable in the present HCC cohort. This difference suggests that the components of the muscle–bone unit may deteriorate at different rates depending on liver-disease etiology, baseline body composition, sex distribution, perioperative course, and postoperative recovery. Several shared biological pathways may explain the coexistence of muscle-quality and bone changes, but they cannot be confirmed from the present retrospective data. Goyal et al. [3] summarized the roles of chronic systemic inflammation, hyperammonemia, anabolic resistance, endocrine dysfunction, malnutrition, accelerated starvation, and physical inactivity in cirrhosis-related muscle impairment. Many of these abnormalities may also influence bone through reduced mechanical loading, impaired vitamin D metabolism, hypogonadism, renal dysfunction, and altered osteoblast–osteoclast activity. Reduced muscle quality may further impair mechanical loading and spinal support, potentially contributing to skeletal vulnerability. Consistent with this concept, Backhauß et al. reported that fatty degeneration of paraspinal muscles was independently associated with non-traumatic vertebral fractures [32]. Monegal et al. [26] demonstrated dynamic postoperative changes in parathyroid hormone, vitamin D, renal function, bone turnover, and bone mass, illustrating that early skeletal remodeling is influenced by multiple interacting pathways. After transplantation, corticosteroid exposure, calcineurin inhibitors, infection, metabolic disturbance, and prolonged inactivity may further affect both tissues. Li et al. [9] reported associations between skeletal muscle density and HDL cholesterol and apolipoprotein A1, suggesting that muscle radiodensity may also reflect systemic metabolic status. Kaul et al. [15] linked malnutrition phenotypes to CT-derived psoas characteristics, reinforcing the possibility that inadequate nutritional recovery contributes to both muscle and bone vulnerability. Clinically, the simultaneous decline in PMD and vertebral attenuation supports coordinated assessment of nutrition, physical function, and bone health instead of evaluating each compartment in isolation.

The present study showed that the most pronounced change occurred during the first six postoperative months, but the temporal patterns of bone and muscle were not identical. Vertebral attenuation declined sharply by POD180 and showed no further measurable reduction by POD365, whereas PMD decreased by POD180; a further decrease by POD365 was observed in the adjusted mixed-effects model but was not significant in the primary analysis after Bonferroni correction. PMA and PMI remained stable throughout follow-up. The early postoperative period includes major surgical stress, reduced mobility, abrupt nutritional and fluid shifts, intensive immunosuppression, infection risk, and endocrine and metabolic disturbance. These exposures may be related to the observed changes, although their individual contributions cannot be determined from the available data. Trabecular bone has relatively high metabolic activity and may respond rapidly to corticosteroid exposure, immobilization, renal dysfunction, and disturbances in calcium–vitamin D–parathyroid hormone regulation. This could explain why vertebral attenuation changed rapidly and then stabilized. In contrast, PMA and PMI depend on the net balance between protein synthesis, catabolism, physical activity, and body-size recovery. Cross-sectional area may therefore remain stable even when contractile tissue is partly replaced by lipid or when intracellular composition deteriorates, potentially explaining the continued PMD decline. Monegal et al. [26] observed an early reduction in bone mass followed by later recovery, whereas Dag et al. [19] found persistent reductions in vertebral attenuation at one year. Elkin et al. [20] reported early PMI loss followed by relative stabilization in recipients with severe obesity and steatotic liver disease, contrasting with the preserved PMI in the present HCC cohort. These differences indicate that postoperative musculoskeletal trajectories are population-specific. Clinically, the first six months may represent the most informative interval for identifying rapid skeletal deterioration, whereas muscle-quality assessment may remain relevant throughout the entire first postoperative year.

The present study suggests that routinely acquired surveillance CT examinations could be used for opportunistic musculoskeletal assessment without exposing recipients to additional radiation or requiring separate imaging appointments. In HCC recipients, abdominal CT is already obtained for oncological, graft, and vascular surveillance, allowing PMD and vertebral attenuation to be extracted from existing examinations. Nachef et al. [33] found that low L1 attenuation was the only independent determinant of prevalent vertebral fracture among cirrhotic LT candidates, indicating that routine CT can identify individuals with clinically relevant skeletal fragility. Mousavi et al. [34] demonstrated a moderate pooled correlation between CT-derived bone measurements and DXA and reported useful discriminatory performance for osteoporosis detection. Dag et al. [16] evaluated LT recipients directly and showed that vertebral and femoral HU values declined progressively across worsening DXA categories, although diagnostic accuracy was not sufficient for CT to replace formal densitometry. These findings support a triage role for vertebral attenuation. For muscle assessment, Li et al. [9] demonstrated prognostic value for skeletal muscle density in HCC recipients, while Huang et al. [24] confirmed the broader association between myosteatosis and post-LT outcomes. Low or declining PMD and vertebral attenuation could therefore prompt more detailed assessment of nutritional intake, muscle strength, physical performance, vitamin D and calcium status, fracture history, and DXA eligibility. Resistance-based exercise, individualized rehabilitation, nutritional optimization, and established bone-protective strategies may be considered according to conventional clinical criteria, but the CT measurements themselves are not yet validated as treatment thresholds. Differences in contrast phase, scanner type, reconstruction algorithm, body composition, and measurement technique limit direct application of universal cutoffs. The present clinical role of these measurements is therefore opportunistic risk stratification and prioritization for further evaluation rather than independent diagnosis or treatment assignment.

### 4.1. Strengths and Limitations

The present study has several strengths that support the validity of its longitudinal interpretation: (i) serial CT measurements were obtained at predefined pretransplant, POD180, and POD365 time points, enabling the timing and persistence of musculoskeletal changes to be evaluated rather than inferred from cross-sectional comparisons; (ii) the use of an age- and sex-matched control group provided an appropriate reference for baseline interpretation; (iii) muscle quantity, muscle quality, and vertebral trabecular attenuation were evaluated simultaneously, allowing their trajectories to be compared within the same recipients; and (iv) hierarchical regression clarified that the crude baseline difference in vertebral attenuation was attenuated after adjustment for age and muscle-related variables, whereas mixed-effects analyses accounted for within-patient correlation and confirmed persistent longitudinal changes in PMD and vertebral attenuation. Nevertheless, several limitations should be acknowledged: (i) the retrospective, single-center design leaves the findings susceptible to residual confounding, precludes causal inference, and may limit generalizability to other transplant populations and imaging protocols; (ii) the relatively small sample size may have reduced statistical power to detect modest longitudinal changes in PMA or PMI; (iii) requiring technically adequate CT examinations at all three time points may have introduced selection bias by preferentially including recipients who survived and remained under structured radiological follow-up; (iv) functional assessments were unavailable, which is relevant because Lepour et al. [25] found the five-times sit-to-stand test to be more strongly associated with outcomes than imaging variables after adjustment, while Arikan et al. [2] showed that sarcopenia defined using both PMI and muscle strength was associated with altered perioperative physiology and adverse postoperative outcomes; (v) concurrent DXA examinations and bone-turnover markers were unavailable owing to the retrospective design and the absence of routine assessments at all predefined pre- and postoperative time points within the institutional follow-up protocol, preventing direct correlation of longitudinal vertebral attenuation changes with densitometric or biochemical measures of bone metabolism; (vi) incident fracture data, cumulative corticosteroid and immunosuppressive exposure, nutritional intake, physical activity, and rehabilitation participation were incompletely available; and (vii) although measurements were obtained using standardized anatomical landmarks, differences in CT scanner characteristics, acquisition parameters, and reconstruction algorithms may have influenced attenuation values. Accordingly, the present findings describe longitudinal associations and temporal patterns rather than causal effects and should be considered hypothesis-generating until confirmed in prospective, multicenter studies incorporating functional, densitometric, biochemical, and treatment-related variables. Different CT systems may have affected HU measurements. Scanner-specific longitudinal data were incomplete, precluding a scanner-restricted sensitivity analysis. Residual inter-scanner variability cannot be excluded. The vertebral levels contributing to the mean attenuation value may have differed between serial examinations when individual levels required exclusion. Potential contributors such as corticosteroid exposure, immunosuppressive regimen, physical activity, rehabilitation, vitamin D status, and fracture events were not systematically available. Therefore, the observed longitudinal changes should be interpreted as associations rather than causal effects of transplantation.

### 4.2. Clinical Perspective and Future Directions

The present study indicates that early musculoskeletal deterioration after LT for HCC may become apparent through declining muscle quality and vertebral trabecular attenuation before overt loss of psoas size. This finding positions PMD and vertebral attenuation as complementary markers to conventional muscle-quantity measurements rather than substitutes for them. Beumer et al. [22] demonstrated the prognostic relevance of low skeletal muscle mass in HCC recipients, Li et al. [9] showed that reduced muscle density provided additional information beyond quantity-based indices, and Sharma et al. [17] reported that low CT-derived bone density was independently associated with poorer post-LT survival. The present study extends these observations by showing that muscle quantity, muscle quality, and bone attenuation do not necessarily change in parallel during the first postoperative year. From a clinical perspective, routine surveillance CT may therefore support integrated opportunistic assessment without additional imaging, although it should not replace DXA, functional testing, fracture assessment, or formal nutritional evaluation. Future prospective multicenter studies should standardize CT acquisition, contrast phase, reconstruction, and segmentation protocols; validate longitudinal changes in PMD and vertebral attenuation against muscle strength, physical performance, DXA, fracture outcomes, bone-turnover markers, nutritional status, and cumulative immunosuppressive exposure; and determine whether CT-guided nutritional, rehabilitation, or bone-health interventions improve clinically meaningful outcomes. The central clinical message is that preserved muscle area does not exclude early postoperative musculoskeletal vulnerability, and serial CT may identify clinically relevant deterioration before conventional quantity-based measures change.

## 5. Conclusions

This study demonstrates that the first postoperative year after LT for HCC is characterized by early deterioration in muscle quality and vertebral trabecular attenuation despite relative preservation of psoas muscle quantity. These findings suggest that muscle quantity, muscle quality, and bone attenuation represent complementary rather than interchangeable dimensions of musculoskeletal health and may follow distinct postoperative trajectories. Routine surveillance CT therefore offers a practical opportunity for integrated opportunistic assessment of both muscle and bone without additional imaging burden, although these imaging biomarkers should currently be regarded as complementary to functional evaluation and established bone health assessment rather than as stand-alone clinical decision-making tools. Prospective multicenter studies incorporating standardized CT protocols, functional performance measures, DXA, bone-turnover biomarkers, and detailed postoperative management data are needed to validate these findings and determine whether longitudinal CT-derived musculoskeletal changes can improve risk stratification and guide targeted interventions after liver transplantation. These findings demonstrate longitudinal associations between the post-transplant period and changes in CT-derived muscle quality and vertebral attenuation but do not establish causality.

## Figures and Tables

**Table 1:** Baseline demographic, laboratory, and CT-derived characteristics of the HCC and control groups.

Variable	HCC Group		Control Group		*p* Value
Age, years	55.4 ± 9.4	56 (52–62)	55.7 ± 9.8	57 (51–63)	0.715 ^m^
Height, cm	171.7 ± 7.1	173 (169–177)	172 ± 8.4	172 (168–179)	0.768 ^m^
Weight, kg	78.4 ± 13.8	77 (70–89)	85.5 ± 15.7	83 (75–98)	0.012 ^t^
BMI, kg/m^2^	26.7 ± 4.8	26 (24–30)	28.7 ± 4.9	29 (25–32)	0.030 ^t^
Albumin, g/dL	3.0 ± 0.7	3.0 (2.5–3.6)	4.0 ± 0.6	4.2 (3.8–4.4)	<0.001 ^t^
Total protein, g/dL	6.9 ± 0.9	7.0 (6.6–7.4)	7.2 ± 0.8	7.3 (6.9–7.7)	0.014 ^m^
Calcium, mg/dL	8.7 ± 0.8	8.8 (8.2–9.2)	9.2 ± 0.8	9.3 (8.9–9.7)	0.001 ^t^
Phosphorus, mg/dL	3.2 ± 1.3	3.0 (2.6–3.6)	3.3 ± 0.7	3.2 (3.0–3.6)	0.149 ^m^
Hemoglobin, g/dL	13.0 ± 2.3	13.3 (11.7–14.6)	14.0 ± 2.3	14.6 (12.6–15.6)	0.011 ^m^
WBC count	5.6 ± 2.7	5.3 (4.2–6.4)	9.6 ± 6.3	7.9 (6.3–10.5)	<0.001 ^m^
Neutrophil count	3.6 ± 2.2	3.2 (2.2–4.2)	8.9 ± 14.5	5.1 (3.7–6.5)	<0.001 ^m^
Lymphocyte count	1.2 ± 0.5	1.2 (0.9–1.6)	2.2 ± 0.9	2.1 (1.6–2.9)	<0.001 ^t^
Platelet count	117 ± 74	103 (71–136)	252 ± 73	245 (201–300)	<0.001 ^m^
RDW	15.3 ± 2.5	14.7 (13.6–16.4)	13.5 ± 1.4	13.2 (12.6–14.0)	<0.001 ^m^
MPV, fL	11.1 ± 1.1	10.9 (10.3–11.7)	10.0 ± 1.0	9.9 (9.2–10.8)	<0.001 ^t^
ALP, U/L	146 ± 83	124 (93–184)	86 ± 47	76 (64–93)	<0.001 ^m^
Baseline L1–4 attenuation	151 ± 36	157 (132–170)	137 ± 39	135 (110–162)	0.037 ^t^
Baseline PMI, cm^2^/m^2^	3.9 ± 1.1	3.9 (3.0–4.6)	4.0 ± 1.4	4.0 (2.8–5.2)	0.634 ^t^
Baseline PMA, cm^2^	11.6 ± 3.3	11.5 (9.2–13.5)	12.1 ± 4.5	12.0 (8.5–15.5)	0.442 ^t^
Baseline PMD, HU	50.5 ± 4.6	51 (48–54)	48.6 ± 6.4	50 (46–52)	0.108 ^m^

Values are presented as mean ± standard deviation and median (Q1–Q3). Continuous variables were compared using ^t^ Student’s *t*-test or the ^m^ Mann–Whitney U test, as appropriate. ALP, alkaline phosphatase; BMI, body mass index; CT, computed tomography; HCC, hepatocellular carcinoma; HU, Hounsfield units; MPV, mean platelet volume; PMA, psoas muscle area; PMD, psoas muscle density; PMI, psoas muscle index; RDW, red cell distribution width; WBC, white blood cell.

**Table 2:** Univariable linear regression analyses of factors associated with baseline L1–L4 vertebral trabecular attenuation.

Predictor	B	95% CI for B	*p* Value	R^2^
Control vs. HCC	−14.094	−27.37 to −0.86	0.037	0.034
Age	−1.301	−1.97 to −0.63	<0.001	0.106
Sex	−11.969	−35.90 to 11.96	0.324	0.008
BMI	0.074	−1.35 to 1.50	0.918	0.000
Albumin	0.604	−7.49 to 8.70	0.883	0.000
Calcium	4.293	−3.81 to 12.39	0.296	0.009
ALP	0.003	−0.09 to 0.10	0.943	0.000
Baseline PMI	9.390	3.93 to 14.85	0.001	0.094
Baseline PMD	1.423	0.24 to 2.60	0.018	0.043

B values, 95% confidence intervals, *p* values, and R^2^ values are shown for crude linear associations with baseline L1–4 attenuation. Each model was estimated using all available observations for the variables included in that specific analysis; therefore, the sample size varied across models according to data availability (*n* = 114–128).

**Table 3:** Hierarchical multivariable linear regression analysis of factors associated with baseline L1–L4 vertebral trabecular attenuation.

Predictor	Model 1 B (*p*)	Model 2 B (*p*)	Model 3 B (*p*)	Model 4 B (*p*)
Control vs. HCC	−16.723 (0.021)	−16.107 (0.022)	−19.098 (0.031)	−10.842 (0.232)
Age	—	−1.375 (<0.001)	−1.436 (<0.001)	−1.187 (0.002)
Sex	—	−1.854 (0.884)	−2.086 (0.869)	15.698 (0.259)
BMI	—	0.499 (0.501)	0.425 (0.570)	−0.389 (0.630)
Albumin	—	—	−3.078 (0.675)	−7.754 (0.286)
Calcium	—	—	6.751 (0.299)	9.084 (0.152)
ALP	—	—	−0.049 (0.336)	−0.022 (0.663)
Baseline PMI	—	—	—	7.654 (0.024)
Baseline PMD	—	—	—	1.090 (0.113)
Model R^2^	0.049	0.175	0.194	0.263
Adjusted R^2^	0.040	0.143	0.138	0.196
ΔR^2^	0.049	0.126	0.019	0.069
*p* for ΔR^2^	0.021	0.002	0.492	0.012
Overall model *p*	0.021	<0.001	0.002	<0.001

Abbreviations: ALP, alkaline phosphatase; BMI, body mass index; PMD, psoas muscle density; PMI, psoas muscle index. Model 1 included study group only; Model 2 additionally included age, sex, and body mass index; Model 3 additionally included albumin, calcium, and alkaline phosphatase; and Model 4 additionally included baseline psoas muscle index and baseline psoas muscle density. All hierarchical multivariable regression models were estimated using the same complete-case sample (*n* = 109). Listwise deletion was applied across all variables included in the final model.

**Table 4:** Multivariate analysis of the baseline CT-derived musculoskeletal profile according to study group.

Analysis	Outcome	HCC	Control	Test Statistic	*p* Value	Partial η^2^
Multivariate test	Overall CT-derived musculoskeletal phenotype	—	—	Pillai’s trace = 0.138; F = 5.238	0.002	0.138
Follow-up univariate test	Baseline L1–L4	149.1	134.0	F = 1.356	0.247	0.013
Follow-up univariate test	Baseline PMI	3.41	2.81	F = 2.542	0.114	0.025
Follow-up univariate test	Baseline PMD	51.5	44.4	F = 14.993	<0.001	0.130

The multivariate model included baseline L1–4 vertebral trabecular attenuation, baseline psoas muscle index, and baseline psoas muscle density as dependent variables. Values for individual outcomes are estimated marginal means adjusted for age, sex, body mass index, albumin, calcium, and alkaline phosphatase.

**Table 5:** Longitudinal changes in CT-derived bone and muscle parameters within the HCC cohort.

Variable	*p*-Value	Effect Size (Partial η^2^)	Baseline vs. POD180	Baseline vs. POD365	POD180 vs. POD365
L1–L4	<0.001	0.462	<0.001	<0.001	1.000
PMI	0.139	0.031	0.286	1.000	0.139
PMA	0.171	0.028	0.396	1.000	0.155
PMD	<0.001	—	<0.001	<0.001	0.020

For L1–4 vertebral trabecular attenuation, the Greenhouse–Geisser-corrected repeated-measures ANOVA result is reported because Mauchly’s test indicated violation of sphericity. For psoas muscle density, the overall longitudinal analysis was performed using the Friedman test, followed by Wilcoxon signed-rank tests for post hoc pairwise comparisons. Because three pairwise comparisons were conducted, statistical significance for post hoc nonparametric comparisons was defined as *p* < 0.0167 after Bonferroni correction.

**Table 6:** Fixed effects from adjusted linear mixed-effects models for longitudinal changes in L1–4 vertebral trabecular attenuation and PMD.

Outcome	Fixed Effect	Estimate (B)	95% CI	F	*p*-Value
L1–L4 (HU)	Time (overall)	—	—	50.761	<0.001
L1–L4 (HU)	Age, per 1 yr	−1.125	−2.024 to −0.226	6.275	0.015
L1–L4 (HU)	BMI	1.156	−0.581 to 2.894	1.775	0.188
L1–L4 (HU)	Albumin	−3.966	−21.888 to 13.957	0.196	0.659
L1–L4 (HU)	Calcium	8.160	−7.182 to 23.503	1.134	0.291
L1–L4 (HU)	ALP	−0.036	−0.142 to 0.070	0.465	0.498
PMD (HU)	Time (overall)	—	—	17.838	<0.001
PMD (HU)	Age, per 1 yr	−0.113	−0.223 to −0.003	4.214	0.045
PMD (HU)	BMI	−0.069	−0.281 to 0.143	0.423	0.518
PMD (HU)	Albumin	1.529	−0.662 to 3.720	1.952	0.168
PMD (HU)	Calcium	−0.301	−2.177 to 1.574	0.103	0.749
PMD (HU)	ALP	−0.005	−0.018 to 0.008	0.618	0.435

Abbreviations: ALP, alkaline phosphatase; BMI, body mass index; CI, confidence interval; HCC, hepatocellular carcinoma; HU, Hounsfield units; PMD, psoas muscle density; POD, postoperative day. Values are derived from linear mixed-effects models fitted in the HCC cohort (*n* = 64), with time entered as a fixed effect and patient identity specified as the repeated subject. Models were adjusted for age, body mass index, albumin, calcium, and alkaline phosphatase. Covariate estimates are unstandardized regression coefficients. Pairwise comparisons are based on estimated marginal means and were adjusted using the Bonferroni method. Positive pairwise mean differences indicate higher values at the earlier time point.

## Data Availability

The data presented in this study are available on reasonable request from the corresponding author. The data are not publicly available due to ethical and privacy restrictions involving human participant data.

## Abbreviations

ALP, alkaline phosphatase; ANOVA, analysis of variance; BMI, body mass index; CI, confidence interval; CT, computed tomography; DXA, dual-energy X-ray absorptiometry; HCC, hepatocellular carcinoma; HU, Hounsfield unit; ICC, intraclass correlation coefficient; LT, liver transplantation; MPV, mean platelet volume; PACS, picture archiving and communication system; PMA, psoas muscle area; PMD, psoas muscle density; PMI, psoas muscle index; POD, postoperative day; RDW, red cell distribution width; ROI, region of interest; STROBE, Strengthening the Reporting of Observational Studies in Epidemiology; WBC, white blood cell.
